# Combined regimen of photodynamic therapy mediated by Gallium phthalocyanine chloride and Metformin enhances anti-melanoma efficacy

**DOI:** 10.1371/journal.pone.0173241

**Published:** 2017-03-09

**Authors:** Diana Tudor, Iuliana Nenu, Gabriela Adriana Filip, Diana Olteanu, Mihai Cenariu, Flaviu Tabaran, Rodica Mariana Ion, Lucian Gligor, Ioana Baldea

**Affiliations:** 1 Department of Physiology, University of Medicine and Pharmacy, Cluj-Napoca, Romania; 2 Department of Biochemistry, University of Agricultural Sciences and Veterinary Medicine, Cluj-Napoca, Romania; 3 Department of Pathology University of Agricultural Sciences and Veterinary Medicine, Cluj-Napoca, Romania; 4 Nanomedicine Research Group, National Institute for Research & Development in Chemistry and Petrochemistry - ICECHIM, Bucharest, Romania; 5 OSRAM Opto Semiconductors, OSRAM Romania, Global City Business Park, Voluntari, Ilfov, Romania; University of Alabama at Birmingham, UNITED STATES

## Abstract

**Background:**

Melanoma therapy is challenging, especially in advanced cases, due to multiple developed tumor defense mechanisms. Photodynamic therapy (PDT) might represent an adjuvant treatment, because of its bimodal action: tumor destruction and immune system awakening. In this study, a combination of PDT mediated by a metal substituted phthalocyanine—Gallium phthalocyanine chloride (GaPc) and Metformin was used against melanoma. The study aimed to: (1) find the anti-melanoma efficacy of GaPc-PDT, (2) assess possible beneficial effects of Metformin addition to PDT, (3) uncover some of the mechanisms underlining cell killing and anti-angiogenic effects.

**Methods:**

Two human lightly pigmented melanoma cell lines: WM35 and M1/15 subjected to previous Metformin exposure were treated by GaPc-PDT. Cell viability, death mechanism, cytoskeleton alterations, oxidative damage, were assessed by means of colorimetry, flowcytometry, confocal microscopy, spectrophotometry, ELISA, Western Blotting.

**Results:**

GaPc proved an efficient photosensitizer. Metformin addition enhanced cell killing by mechanisms dependent on the cell line, namely apoptosis in the metastatic M1/15 and necrosis in the radial growth phase, WM35. Cell death mechanism relied on the inhibition of nuclear transcription factor (NF)-κB activation and tumor necrosis factor (TNF)—related apoptosis-inducing ligand (TRAIL) sensitization, leading to TRAIL and TNF-α induced apoptosis. Metformin diminished the anti-angiogenic effect of PDT.

**Conclusions:**

Metformin addition to GaPc-PDT increased tumor cell killing through enhanced oxidative damage and induction of proapoptotic mechanisms, but altered PDT anti-angiogenic effects.

**General significance:**

Combination of Metformin and PDT might represent a solution to enhance the efficacy, leading to a potential adjuvant role of PDT in melanoma therapy.

## Introduction

Melanoma is a malignant tumor derived from melanocytes with one of the most rapidly increasing incidence in the world. In the past 50 years the mortality has also increased, without any clear path to melanoma prevention [1]. Melanoma registered globally approximately 160 000 new cases and 48 000 deaths/year [2]. Once distant sites from the skin become seeded, melanoma becomes one of the most aggressive tumors, with a life expectancy lower than 12 months. Many treatment strategies like: surgery, chemotherapy, radiotherapy, BRAF and mitogen-activated protein kinase (MAPK) pathway inhibitors, immunotherapy and anti-angiogenic therapies are used related to the stage of the disease. However, tumor resistance mechanisms hinder the efficacy of therapy; therefore future approaches need to focus on this direction. One possible solution might be the old molecule Metformin, due to the inhibition of the stemness character of melanoma cells [3]. Metformin is used as a hypoglicemiant drug in type 2 diabetes mellitus and lately became a promising drug in oncology. Retrospective studies revealed decreased cancer incidence and cancer-related mortality in obese and diabetic patients treated with Metformin [4]. Metformin triggers antitumor activity in several cancers (e.g. lung, breast, prostate and pancreas) [5]. In melanoma, Metformin was shown to induce cell death and arrested melanoma invasion and metastasis, via pro-apoptotic mechanisms [6]. In anti-melanoma therapy there are three ongoing clinical trials that are recruiting patients and are using Metformin in combination with BRAF inhibitors (ClinicalTrials.gov, Identifier: NCT01638676 and NCT02143050) and also in association with Dacarbazine (ClinicalTrials.gov, Identifier: NCT02190838). In a previous study conducted by our group, association of Metformin to PDT in Walker-256 carcinosarcoma experimental model improved the overall anti-tumor effects [7]. Based on these findings, the current research aims to study the possible anti-tumor role of Metformin as an adjuvant in photodynamic therapy against melanoma.

Photodynamic therapy (PDT) is a two steps oncological therapy: (1) administration of a photosensitizer (PS) (2) and tumor irradiation by light of a specific wavelength [8]. Light activation of the PS generates reactive oxygen species (ROS) in the targeted tumor area [9], that destroy tumor cells through cell death induction, destruction of tumor vessels and activation of an immune response [10].

Thus, PDT might be considered an ideal anticancer therapy, because of the primary tumor destruction and also immune activation. This immune reaction should be able to track down and destroy any remaining tumor cells of the primary tumor or distant micro metastases [11]. However, melanoma may be often resistant to PDT. The most important resistance mechanisms are: presence of melanin that absorbs PDT light and has an anti-oxidant effect, sequestration of the PS into melanosomes, apoptotic pathways errors and antioxidant defense that eventually lead to further tumor development [12]. Recent studies gave a new hope by providing encouraging PDT strategies to overcome the aggressiveness of melanoma [13, 14]. These strategies include finding new PS molecules, able to accumulate within tumor cells and to generate enough ROS upon light activation to overcome the resistance of melanoma cells. One group of these photosensitizers is represented by phthalocyanines (Pc). Phtalocyanines are macrocyle compounds activated by the same light wavelengths as porphyrins. Pc are second-generation PS that exhibit important effective tissue penetration due to their chemical stability, high yields of ROS generation and good spectroscopic properties [15]. These make them more suitable in melanoma PDT since they might overcome the melanoma defense. Unfortunately, there is insufficient data regarding their applicability in oncology. In anti-melanoma therapy one report states that aluminium tetrasulfophthalocyanines used at a photosensitizing concentration of 40 μg/mL in combination with a light dose of 4.5 J/cm^2^ induced melanoma cell death [16]. Gallium phthalocyanine (GaPc), indium (III) and iron (III) phthalocyanine chloride at a concentration of 2 μg/mL were potently phototoxic towards lung cancer cells in vitro upon light regimen exposures of 2.5 J/cm^2^, 4.5 J/cm^2^ and 8.5 J/cm^2^[17].

The present study evaluates the antitumor effects induced by the combined regimen of Gallium phthalocyanine chloride mediated-PDT with Metformin, used as an adjuvant, on two human lightly pigmented melanoma cell lines with different stages of development and aggression: a radial growth phase (WM35) and a metastatic cell line (M1-15), with focus on different mechanisms involved in oxidative stress induced cellular death, angiogenesis and inflammation.

## Materials and methods

### Chloro-gallium (III) phthalocyanine synthesis and characterisation

**Fig 1:**
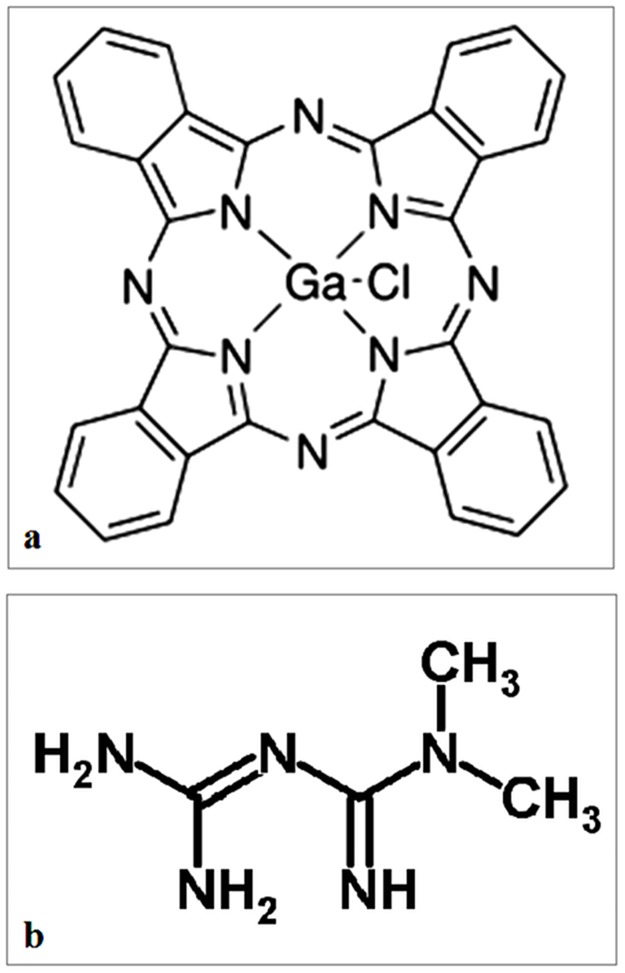
Chemical formula. Chloro-gallium (III) phthalocyanine (a) and Metformin (b).

The preparation of Chloro-gallium (III) phthalocyanine (GaPc) was achieved, following a previously reported method [18]. Phthalonitrile (2.00 g, 0.72 mmol) and GaCl_3_ (0.315 g, 0.27 mmol) were placed in a preheated oil bath (215°C); after that 1-chloronaphthalene (0.35 mL) has been added. The reaction mixture was refluxed under dry inert gas (N_2_) for 1.5–2 h at the same temperature. After cooling, the product was added to methanol; the precipitate was filtered, washed intensely with methanol and acetone, and dried in vacuum. The products were purified by elution with CHCl_3_ through a short column (≈ 5 cm) of Al_2_O_3_ (Degree 3). Yield: 0.75 g (23%). Chemical formula is presented in Fig 1. UV/Vis (DMSO): λmax (ε) / (nm/M-1.cm-1); 680 (138038), 645 (83170), 610 (10000), 355 (26300). IR (KBr): νmax/cm-1; 1500 (C = C); 1H NMR (DMSO-d6) δ, ppm; 9.80 (8H, d, Pc), 8.60 (8H, d, Pc). MS: m/z = 1059.80. GaPcCl was solubilized in dimethylsulfoxide (DMSO) to prepare a stock solution of 10 μg/ml that was further used to prepare the final solutions in medium immediately before use for cell treatment. The DMSO final concentration in the medium was <0.05%, not toxic to the cells [19].

### Melanoma bioassays

#### Cell cultures

The assessment was performed on radial growth phase-WM35 human melanoma (Wistar Institute, Philadelphia, PA, USA) [20] cells and a highly metastatic melanoma cell line M1-15 [21], donated by professor Andras Falus, Genetics Department, University Semmelweis, Budapest. Melanoma cells were cultured in RPMI medium supplemented with 5% fetal calf serum, 50 μg/ml gentamicin and 5 ng/ml amphotericin, all from Biochrom AG (Berlin, Germany), to avoid the medium influence on the cell lines properties, as previously described [22, 23]. For all experiments, the cells were used within 4 passages, to preserve their original melanoma characteristics [24, 25]. Cultures were fed twice weekly and incubated in a humid atmosphere at 37°C and 5% CO_2_. All experiments were conducted in triplicate in subdued light, as previously described [26].

#### Light source

PDT irradiation was done by a red light lamp obtained from OSRAM Opto Semiconductors, Bucharest, Romania (wave length 630 nm, lamp power 11.83 mW/cm^2^, measured at a distance of 5 cm from the lamp) with doses of 2.5 J/cm^2^ and respectively 5 J/cm^2^.

#### Cytotoxicity assay

The cells were seeded at a density of 10^4^/well in ELISA 96 wells micro titration flat bottom plaques and allowed to settle for 24 h. Then cells were exposed for 24 h either to Metformin chloride (8 mM) (Sigma Chemical Co., St. Louis, MO, USA) solved in medium [27] or medium, washed then treated with GaPc, prepared as described above, in concentrations ranging from: 1–2000 μg/ml (1, 10, 25, 50, 100, 200, 500 and 1000) in medium for 24 h. Cells were then washed, afterwards irradiated with 2.5 J/cm^2^ and 5 J/cm^2^ and further incubated for 24 h with fresh medium. Experiments using single Metformin exposure in doses ranging from: 0–128 mM (0, 2, 4, 8, 16, 32, 128) were also performed. These cells were then exposed to irradiation in a dose of 2.5 J/cm^2^ to quantify the combined Metformin irradiation effect on cell viability. Viability was measured by colorimetric measurement of formazan, a coloured compound generated by mitochondrial reductase activity in viable cells using CellTiter 96^®^ AQueous Non-Radioactive Cell Proliferation Assay (Promega Corporation, Madison, WI 53711 USA), as indicated by the producer, readings were done using an ELISA plate reader at 540 nm (Tecan, Männedorf, Switzerland). Untreated cultures exposed to medium were used as controls. Cytotoxicity is presented as OD 540. Pictures were taken through an inverted microscope (Olympus CKX 41, Hamburg, Germany), using a digital camera (Olympus, E 330) and original magnification 10 times.

#### Experimental design

Melanoma cells (WM35 and M1-15) seeded in Petri dishes at a density of 10^4^/cm^2^ were exposed to either Metformin, or GaPc, or GaPc with previous Metformin treatment, untreated cells were used as controls. For confocal microscopy studies, WM35 cells were seeded on chamber slides, at a density of 5x10^3^/cm^2^ (Nalgene, Rochester, NY, USA). Following the different exposure regimens, cells were washed, further incubated for 24 h with medium and afterwards tested for cell death induction (Annexin-FITC/PI staining- flowcytometry, confocal microscopy, TNF—related apoptosis-inducing ligand—TRAIL, ELISA), oxidative stress induced damage and alterations like (malondialdehyde—MDA, nitric oxide (NO) formation spectrophotometry, NF-κB activation, WB), melanogenesis (total melanin content spectrophotometry, tyrosinase protein, microftalmia transcription factor -MITF, WB) inflammation (tumor necrosis factor α -TNF-α, ELISA), cytoskeleton alterations (phalloidin staining, confocal microscopy) and also angiogenesis [vascular endothelial growth factor—VEGF, ELISA; hypoxia inducible factor (HIF)-1α, WB]. The melanin content of the cells was low and had no influence on the methods used for quantification. Moreover, the same method was used for control and treated groups.

#### Cell death mechanism

For the assessment of cell death mechanism, treated cells (as described in section 2.2.4) were stained with Annexin V-fluorescein isothiocyanate (FITC)/vital dye propidium iodide (PI) (BD Pharmingen Biosciences, San Jose, CA, USA). Viable cells were Annexin V (-)/ PI (-), early apoptotic cells were identified as Annexin V-FITC positive (green) cells, while necrotic cells were PI positive (red fluorescence), late apoptosis was shown by Annexin V (+)/PI (+). Differentiation among these cell populations was done by flow cytometric detection using a BD FACS Canto II flow cytometer (Becton Dickinson & Company, Franklin Lakes, NJ, USA) equipped with two lasers as excitation sources: blue (488 nm, air cooled, 20 mW solid state) and red (633 nm, 17 mW HeNe), as previously described [28]. For confocal microscopy assessment of cell death, cells, treated as above (section 2.5) were stained with Annexin V-FITC/PI, according to the manufacturer’s instructions and then fixed in 2% paraformaldehyde.

#### Cytoskeleton morphology

Phalloidin—FITC 50 μg/ml (Sigma Chemical Co., St. Louis, MO, USA) a marker for the actin miofilaments (green) was used in combination with DRAQ5 (Sigma) staining (red) for nuclei. Images were recorded using a 63 times oil immersion apochromat Zeiss objective (Zeiss LSM 710 Confocal Laser Scanning unit, Carl Zeiss AG, Oberkochen, Germany). For the annexin—FITC and Phalloidin—FITC excitation/emission of 490/525 nm, for PI a 630/680 nm excitation/emission, for DRAQ5 a 646 nm excitation/detection at 681 nm was used. Image combining, processing, and analysis were performed using the standard ZEN software package (Carl Zeiss MicroImaging GmbH, Oberkochen, Germany) [29].

#### Melanogenesis assessment

Total melanin content was determined through spectrophotometry (Sigma) as previously described [19]. Data were expressed as mg/ml. The enzymatic activity of tyrosinase, as DOPA oxidase, was determined through spectrophotometry as previously described [19, 30]. Data were expressed as Units/mg protein. All reactive were purchased from Sigma.

#### Inflammatory and neoangiogenesis markers

Were assessed by ELISA and Western Blot (WB). TRAIL, VEGF and TNF-α ELISA Immunoassay kits from R&D Systems, Inc (Minneapolis, USA) were used. Melanoma cells supernatants were treated according to manufacturer’s instructions; readings were done at 450 nm with correction wavelength set at 540 nm, using an ELISA plate reader (Tecan).

The cell lysates used for the following determinations were prepared as previously described [31]. Protein concentrations were determined by the Bradford method according to the manufacturer’s specifications (Biorad, Hercules, California, USA) and using bovine serum albumin as standard. For all assays the lysates were corrected by total protein concentration. For Western Blotting, lysates (20 μg protein/lane) were separated by electrophoresis on SDS PAGE gels and transferred to polyvinylidenedifluoride membranes, using Biorad Miniprotean system (BioRad, Hercules, Califormia, USA). Blots were blocked and then incubated with antibodies against: NF-κB, phospho- pNF-κB p65 (Ser536) (93H1) (pNF-kB), IκKα, IκKβ (L570) (IP Preferred), phospho-IκK α/β (Ser176/180) (16A6) (pIκK α/β) (Cell Signaling Technology, Inc, Danvers, USA), HIF1α, tyrosinase and MITF (Santa Cruz Biotechnology, Delaware Ave, Santa Cruz, USA) then further washed and incubated with corresponding secondary peroxidase-linked antibodies (Santa Cruz Biotechnology). Proteins were detected using Supersignal West Femto Chemiluminiscent substrate (Thermo Fisher Scientific, Rockford IL, USA), and a Gel Doc Imaging system equipped with a XRS camera and Quantity One analysis software (Biorad). GAPDH (Trevigen Biotechnology Gaithersburg, MD, Maryland, USA) was used as a protein loading control [24, 25].

#### Oxidative stress-induced damage

To investigate whether PDT can induce oxidative stress, quantification of malondialdehyde (MDA) a marker for the peroxidation of membrane lipids and nitric oxide (NO) formation were performed by spectrophotometry, as previously described. All reactives were purchased from Sigma. Data were expressed as nM/mg protein [32].

### Statistical method

Statistical significance of the difference between treated and control group was evaluated by the two-way ANOVA, TTEST and Tukey Posttests. *p* value less than 0.05 was considered to represent a statistically significant difference. Inhibitory concentration 50% (IC50) was calculated for each cell line and irradiation dose. Statistical package Prism version 6.00 for Windows, GraphPad Software, San Diego, California, USA, www.graphpad.com was used for data analyses.

## Results

### Cell viability

**Fig 2:**
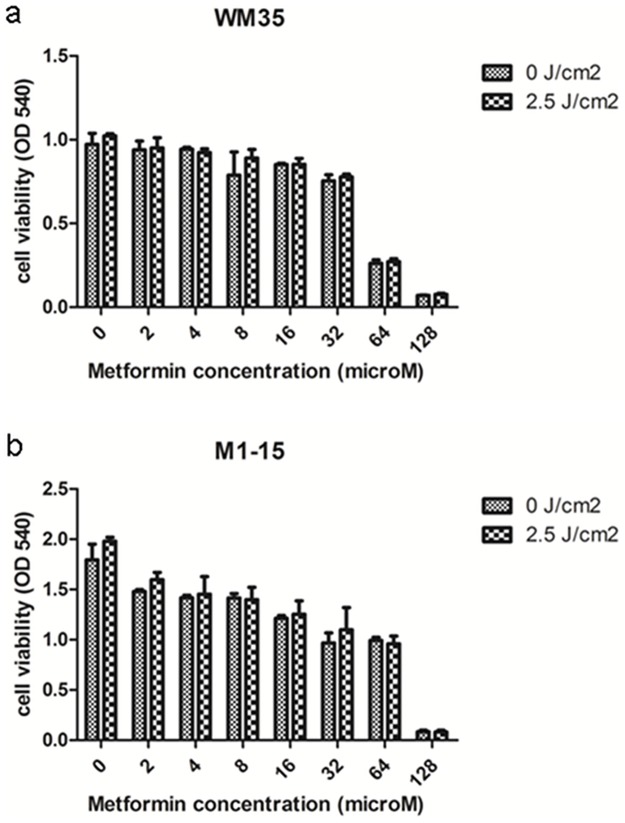
Viability testing after Metformin exposure. Melanoma cell cultures exposed to different concentrations of Metformin or Metformin and irradiation (a)WM35 and (b)M1-15. OD 540 graphs were generated using GraphPad Software and show mean values ± standard deviation, n = 3 for each sample. Cell viability of both cell lines was decreased by increasing concentrations of Metformin, in a dose dependent manner; irradiation had no effect on the cell viability.

**Fig 3:**
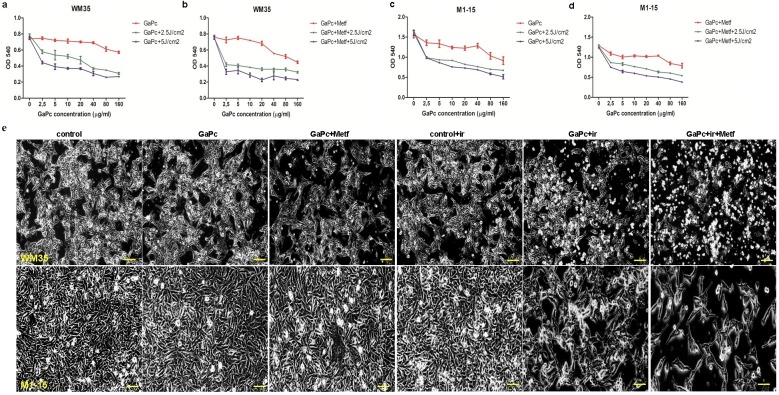
Viability testing after GaPc-PDT and Metformin exposure. WM35 (a, b), M1-15 (c, d) melanoma cell cultures exposed to GaPc-PDT (a, c), respectively GaPc-PDT+ Metformin (b, d). OD 540 graphs were generated using GraphPad Software and show mean values ± standard deviation, n = 3 for each sample. Cell viability of both cell lines was decreased by GaPc-PDT depending on GaPc concentration and irradiation dose; Metformin increased the efficacy of GaPc-PDT especially in WM35 cells. e-images of WM35 (upper panels) and M1-15 (lower panels) cells subjected to GaPc-PDT and Metformin. Cells exposed to GaPc w/o Metformin, showed a normal morphology, compared to controls, while PDT irradiation of treated cells induced loss of cell adhesion, pleiomorphysm with spherical or bipolar shaped cells, signs of treatment induced photo-toxicity.

Viability was quantified by colorimetry (Figs 2 and 3). In WM35 melanoma, GaPc-DT reduced viability in a dose dependent manner (IC50 = 26.98 μg/ml at 2.5 J/cm^2^ and 20.11 μg/ml at 5 J/cm^2^) with no dark toxicity (IC50 = 161.5 μg/ml) at effective doses (Fig 3).

Metformin induced a dose dependent decrease in cell viability in both cell lines (Fig 2), independent of irradiation exposure (for WM35, IC50 = 49.5 mM and IC50 = 51.1 mM when Metformin exposure was followed by 2.5 J/cm^2^ irradiation; for M1-15, IC50 = 28.9 mM and IC50 = 29.5 mM with 2.5 J/cm^2^ irradiation). When only Metformin was used, the concentration of 8 mM induced a slight, not significant decrease of the viability for the metformin treated cells (p≤ 0.061724) compared to the controls. Therefore, a concentration of 8 mM Metformin, lower than therapeutically relevant plasma concentration of Metformin (20 mM/L) was further used for the PDT combined treatment [33].

Combination treatment of GaPc PDT with Metformin increased dark toxicity (IC50 = 60.22 μg/ml) and phototoxic effects of PDT with IC50 = 11.82 μg/ml at 2.5 J/cm^2^ and 6.07 μg/ml at 5 J/cm^2^. In the M1-15 metastatic melanoma line, PDT showed a similar pattern of viability decrease, dependent on the light dose (IC50 = 21.71 μg/ml at 2.5 J/cm^2^ and 12.18 μg/ml at 5 J/cm^2^), without dark toxicity (IC50 = 316.35 μg/ml). Improvement of the phototoxic effect due to Metformin addition was discreet (IC50 = 19.56 μg/ml at 2.5 J/cm^2^ and 11.42 μg/ml at 5 J/cm^2^), without increasing the dark toxicity of GaPc (IC50 = 327.3μg/ml). Based on these data, the following experiments were done using the GaPc concentration of 7.5 μg/ml and 10 μg/ml for ELISA measurements. PDT light dose was 2.5 J/cm^2^.

### Cell death mechanism

**Fig 4:**
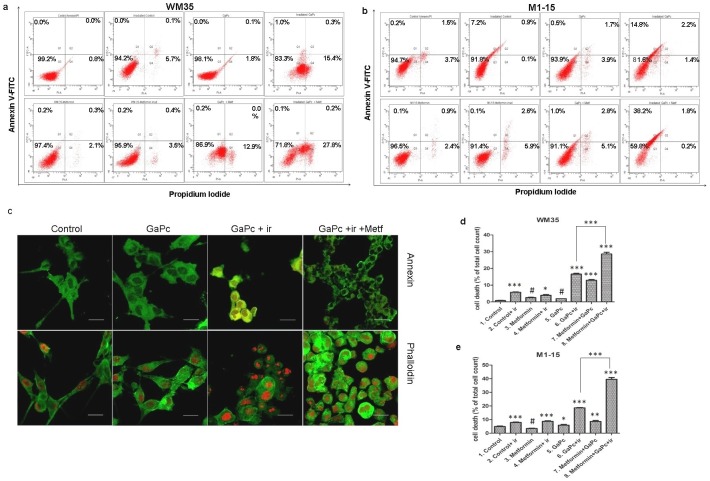
Cell death assessment. Comparative FACS analysis following GaPc-PDT + Metformin treatment versus controls in WM35 (a) and M1-15 (b) cells; c- confocal microscopy images of treated WM35 cells stained with annexin V-FITC/ PI (upper panels) and phalloidin-FITC and DRAQ5 (lower panels), original magnification 63x; PDT exposed cells showed annexin V positive (green) and some exhibit PI positive red fluorescence, while Metformin addition increased the number of annexin V/PI positive cells, there are also present stress related morphological changes; phalloidin staining showed cytoskeleton alterations like increased condensations of actin filaments, retraction of dendrites, spherical shaped cells and loss of cell adhesion in PDT w/o Metformin treated cells. d, e quantitative FACS results for WM35 (d) and M1-15 (e) are expressed as % of total dead cells—annexin V and PI positive cells, from the total cell number; ir = irradiated cells; # = not significant, * = p<5.0E-02, ** = p<1.0E-02, *** = p<1.0E-03. Each bar represents mean ± standard deviation (n = 3).

Cell death mechanism was assessed by flowcytometric analysis and confocal microscopy following annexin/PI staining of treated cells with 7.5 μg/ml GaPc (Fig 4). In both cell lines, GaPc showed no significant cell death induction in the absence of irradiation. A high percentage of cell death was obtained in the GaPc-PDT group, with a significant increased cell death from 15.8% to 28.1% for WM35 and from 18.4 to 40.2% for M1-15, when Metformin was added. The main cell death mechanism was necrosis for WM35 and apoptosis for the M1-15 metastatic cell line. Metformin exposure, followed by irradiation increased tumor cell killing in the M1-15 (9.6%), while in the WM35 melanoma cells, it produced no significant effect on cell death. Combination with GaPc potentiated the antitumor effect in both cell lines. These results show that in both melanoma cell lines, Metformin effectively increased PDT induced cell death. This effect is leading to a better therapeutic response overcoming the melanoma activation of survival mechanisms. Moreover, tumor killing was not influenced by the melanoma stage.

### Cytoskeleton alterations

PDT alterations of the cytoskeleton were seen through confocal microscopy techniques, following phalloidin staining of the actin filaments (Fig 4c). Exposure to GaPc without irradiation did not induce any cytoskeleton alterations. However, in both cell lines exposed to PDT, the actin filaments exhibited condensations, disrupted microtubule network, retraction of dendrites, polymorphism with spherical shaped cells and loss of cell adhesion.

### Oxidative stress damage

**Fig 5:**
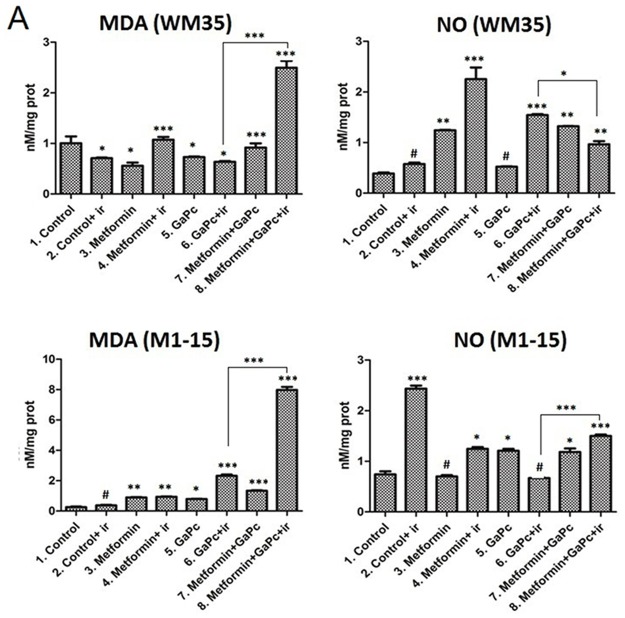
Oxidative stress assessment. Malondyaldehide (MDA), nitric oxide (NO) levels (nM/mg protein) measurements in WM35 (upper panels) and M1-15 (lower panels) were done by spectrophotometry. Each bar represents mean ± standard deviation (n = 3). ir = irradiated cells; # = not significant, * = p<5.0E-02, ** = p<1.0E-02, *** = p<1.0E-03.

In order to quantify the oxidative stress and respectively nitrozative stress induced by PDT and combined treatment, MDA, a marker of lipid peroxidation and NO formation were measured (Fig 5).

#### Malondyaldehide

In both cell lines, GaPc and the combination of GaPc and Metformin without irradiation slightly increased lipid peroxidation. However, MDA levels were significantly elevated (from ~ 0.9 nM/mg protein to 2.6 nM/mg protein in WM35 and from 0.26 nM/mg proteins to 7.9 nM/mg proteins in M1-15) in cells subjected to the combined GaPc-PDT and Metformin therapy, as compared to controls. Moreover, Metformin addition proved beneficial to the oxidative stress induced damage in the treated melanoma cells, compared to GaPc-PDT.

#### Nitric oxide formation

In both cell lines, NO was increased with irradiation. Metformin addition had a different effect in the melanoma cells. In WM35, Metformin significantly increased the NO, while in the M1-15, it diminished the NO formation and both effects were enhanced by irradiation. When GaPc-PDT was used, NO was highly increased in the WM35 from 0.38 nM/mg protein to 1.54 nM/mg protein. In M1-15 the NO generation, following PDT was not significant. Metformin addition to GaPc-PDT increased NO levels only in M1-15, compared to GaPc-PDT.

#### TNF-α and TRAIL expression

**Fig 6:**
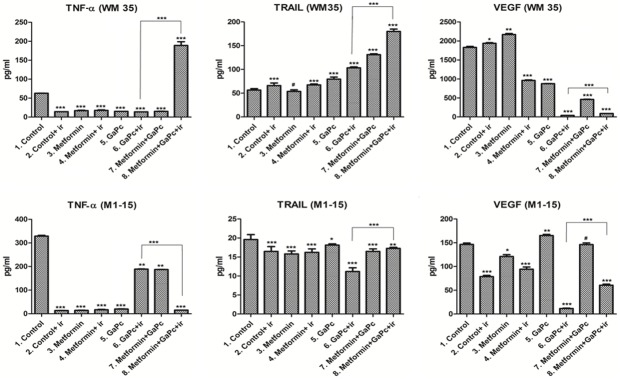
Assessment of inflamatory and angiogenetic markers. Protein expressions of TNF-α, TRAIL, VEGF (pg/ml) in melanoma cultures exposed to GaPc-PDT + Metformin were determined by ELISA, upper panels (WM35), lower panels (M1-15); Each bar represents mean ± standard deviation (n = 3), ir = irradiated cells; # = not significant, * = p<5.0E-02, ** = p<1.0E-02, *** = p<1.0E-03.

To assess the inflammation and its role in the induction of cell death, the levels of TNF-α and the TNF-related apoptosis-inducing ligand were measured by means of ELISA. In the WM35 line, GaPc-PDT and Metformin highly increased TNF-α levels, as well as TRAIL expression, compared to all other groups. This suggests that association of Metformin with GaPc-PDT induced efficient pro-inflammatory and pro-apoptotic responses (Fig 6). Moreover, the high levels of the inflammatory molecules were correlated with oxidative stress (MDA level) and tumor cell death. This effect was different in the case of M1-15. In the untreated M1-15, cells surprisingly secreted high levels of TNF-α, compared to the therapeutical regimen GaPc-PDT and Metformin. This finding emphases the intricate role of TNF-α in cancer. In the metastatic cell line, TNF-α might have a pro-tumoral effect, which explains the high levels in the controls, helping the cells evade from the apoptotic mechanisms. However, TNF-α level was strongly inhibited by the GaPc-PDT and Metformin and this was combined with a high expression of TRAIL, leading to an increased apoptotic cell death. To further study the effect of the therapy induced oxidative stress; we measured, by means of Western Blot, NF-κB activation.

#### NF-κB activation

In order to assess the role of the NF-κB pathway activation in the survival of the melanoma cells treated with PDT, western blot was performed to quantify the protein levels of the total NF-κB protein and the active form, pNF-κB—p65 (at Ser 536) and also the IκKα, IκKβ and the phosphoryted pIκK. Phosphorylation of p65/RelA at Ser 536 regulates activation, nuclear localization, protein-protein interaction and transcriptional activity. The key in the NF-κB pathway activation involves a high molecular weight Ikappa Kinase (IκK) complex, consisting of three associated IκK subunits. IκKα, and IκKβ serve as the catalytic subunits of the kinase. Activation of the IκK depends on the phosphorylation at Ser 177 and Ser 181 in the activation loop of IκKβ and Ser 176 and Ser 180 in IκKα.

**Fig 7:**
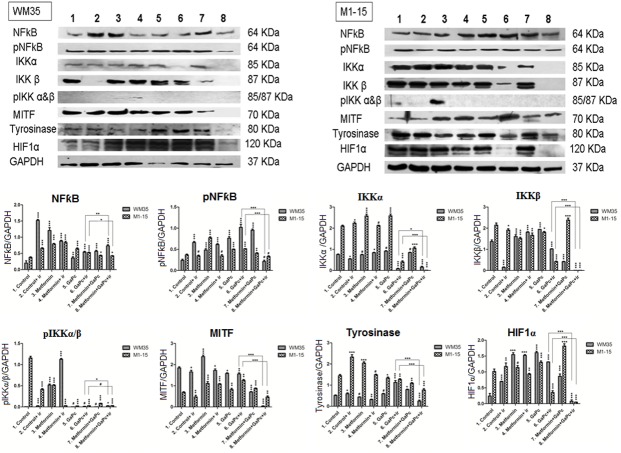
Protein expression measured by Western Blot. Protein expressions of NF-κB, pNF-kB, IκKα, IκKβ, pIκK α/β, HIF1α, tyrosinase and MITF in melanoma cells treated with GaPc-PDT + Metformin, left panel (WM35), right panel (M1-15) were measured by WB. Image analysis of WB bands was done by densitometry, results were normalised to GAPDH.—WB images (upper panels) 1 = control, 2 = irradiated control, 3 = Metformin, 4 = irradiated Metformin, 5 = GaPc, 6 = GaPc-PDT, 7 = GaPc and Metformin, 8 = GaPc-PDT and Metformin; graphical representation of quantitative WB results for WM35 and M1-15 (lower panels); ir = irradiated cells; # = not significant, * = p<5.0E-02, ** = p<1.0E-02, *** = p<1.0E-03. Each bar represents mean ± standard deviation (n = 3).

In WM35, irradiation, Metformin exposure and GaPc-PDT increased the expression of NF-κB and pNF-κB, compared to controls. Furthermore, there was a significant increase in NF-κB protein expression induced by GaPc-PDT and Metformin compared to GaPc-PDT (Fig 7). However, only a modest part of NF-κB protein was represented by the active form. In this respect, a higher amount of pNF-κB was obtained when cells were exposed to PDT alone, suggesting that Metformin addition to these cells increased the total NF-κB protein, but decreased NF-κB activation, a mechanism involved in survival of the melanoma cells leading to resistance to PDT. In the M1-15 melanoma, NF-κB protein was significantly increased following irradiation, Metformin and GaPc single exposure and respectively GaPc-PDT, compared to controls. When Metformin was added to GaPc-PDT, NF-κB decreased compared to GaPc-PDT. pNF-κB levels were higher when cells were treated with GaPc and GaPc-PDT. Metformin and GaPc-PDT decreased pNF-κB at control levels.

In both cell lines, IκK complex proteins were increased in cells exposed to Metformin and GaPc, while PDT strongly inhibited both of them, leading to extremely low levels in Metformin and GaPc-PDT groups. The active pIκK form was strongly inhibited in the last four groups, when GaPc was used. This shows that even though there was a stimulation of the NF-κB protein following GaPc-PDT, the activation pathway was strongly inhibited by the combined Metformin and GaPc-PDT treatment.

### Melanogenesis

**Fig 8:**
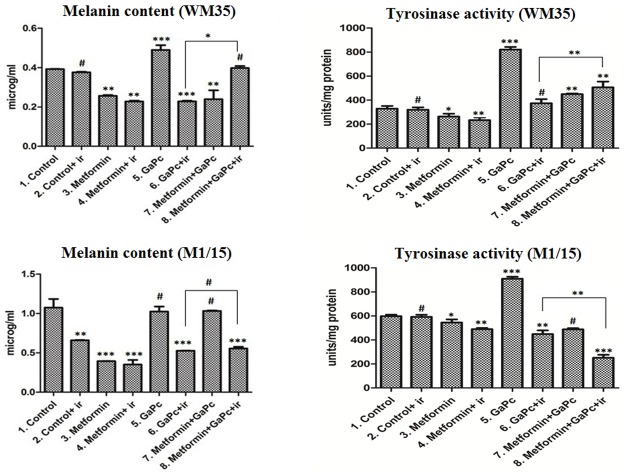
Melanogenesis. Total melanin content (μg/ml) and tyrosinase enzymatic activity (Units/mg protein) measurements in WM35 (upper panels) and M1-15 (lower panels) were done by spectrophotometry. Each bar represents mean ± standard deviation (n = 3). ir = irradiated cells; # = not significant, * = p<5.0E-02, ** = p<1.0E-02, *** = p<1.0E-03.

To assess the influence of the PDT and Metformin on melanogenesis in the melanoma cells, the total melanin content and the enzymatic activity of tyrosinase (Fig 8), as DOPA oxidase were measured by spectrophotometry. Expression levels of tyrosinase, the key melanogenic enzyme, and the microftalmia transcription factor—MITF, involved in melanogenesis regulation and also survival, differentiation and resistance to PDT were also quantified by means of Western Blotting (Fig 8).

#### Melanin content

As expected, the melanin content was low, since both these cell lines are lightly pigmented. Exposure to GaPc enhanced melanin content, while PDT exhibited a strong inhibitory effect in both cell lines (Fig 8). Metformin and GaPc-PDT induced a melanogenic level similar to the control in WM35 cells while in the M1-15, there was no significant difference between the PDT groups.

#### Tyrosinase

Tyrosinase activity correlated with the levels of melanin (Fig 8). PDT inhibited the tyrosinase activity in both cell lines, while combined treatment only decreased tyrosinase activity in M1-15 cells. In WM35 melanoma, the tyrosinase protein level was increased by GaPc-PDT compared to controls, while Metformin and GaPc-PDT decreased tyrosinase, compared to controls. In M1-15 cells, GaPc-PDT decreased the tyrosinase, and the combined regimen enhanced this effect (Figs 7 and 8).

#### MITF

MITF levels were inhibited in WM35 cells by PDT exposure and Metformin potentiated this effect, when used in combination with PDT (Fig 7). Metformin exposure increased MITF expression in both cell lines. In M1-15, MITF protein was enhanced following GaPc-PDT, while Metformin and GaPc-PDT decreased MITF, compared to controls. Overall, melanogenesis was decreased by PDT in the metastatic M1-15 cell line and this effect was enhanced by combined treatment. In WM35 the effects were different. PDT increased melanogenesis. Addition of Metformin strongly inhibited the protein levels of tyrosinase and MITF and decreased melanin and tyrosinase activity compared to PDT alone. The inhibition exerted by the combined treatment on MITF expression was seen in both cell lines.

### Neoangiogenesis markers

To assess the possible anti-angiogenic effects of the PDT experimental setting, the hypoxia inducible factor 1 (HIF-1α), involved in carcinomatous neoangiogenesis formation and it’s target, the main neoangiogenesis stimulator, vascular endothelial growth factor (VEGF) were measured by means of Western Blotting and ELISA (Figs 6 and 7).

#### HIF-1α

In WM35 cells, HIF1-α was increased by Metformin and GaPc treatment, and also by GaPc-PDT. When Metformin was combined with GaPc-PDT, HIF-1α was strongly inhibited (Fig 7). In the M1-15 cell line, HIF-1α basal level was increased. Metformin and GaPc had almost no effect on the protein expression. PDT diminished HIF-1α compared to controls and the combined treatment had a stronger effect.

#### VEGF

In both melanoma cell lines, GaPc-PDT and GaPc-PDT associated with Metformin showed an important antiangiogenic activity in contrast with the high levels of VEGF in the untreated cells (Fig 6). However, the combined treatment had a lower inhibitory effect on VEGF compared with GaPc-PDT alone. Overall, PDT inhibited neoangiogenetic promoting factors in both cell lines. This effect was stronger with the combined treatment in the case of HIF-1α.

## Discussion

The present research investigates the possible benefits of using Metformin as an adjuvant in PDT against two human melanoma cell lines with different degrees of aggression: WM35, a radial growth phase line and M1-15, a metastatic melanoma line, with focus on cell death, oxidative stress induced damage, inflammation and angiogenesis.

In addition, we also studied the efficiency of Gallium phthalocyanine chloride (GaPc) as a photosensitizer in PDT against melanoma. In a previous study, we found good PDT efficacy against WM35 melanoma cells using as PS an Indium substituted Pc: chloride indium (III) Pc [ClIn (III)Pc], activated by red light at a dose of 6 J/cm^2^ [34].

In our study, GaPc decreased viability in both melanoma cell lines, in a dose related manner, without dark toxicity. PDT efficiency was increased in the case of WM35. This is consistent with results presented by others that found GaPc to be an efficient photosensitiser, in different cancers such as: colon cancer Caco-2 cells or lung cancer cells [35, 35, 36] Although, we found no evidence of GaPc-PDT assays in melanoma, another Pc used for PDT, aluminum tetrasulfophthalocyanine showed efficient melanoma cells killing. Pc’s properties can be improved by incorporating metals such as gallium, zinc and aluminium [17, 36]. Metallation of PCs with diamagnetic ions: Zn ^2+^, Al ^3+^, and Ga ^3+^, was reported to increase photosensitiser quantum yields and lifetimes (ΦT 0.56, 0.50 and 0.34 and *τ*T 187, 126 and 35 *μ*s) [37, 38]. This potentially increases the photosensitiser capabilities. However, the problem of tumor delivery still remains, mainly due to their lipophylic character, poor tumor specificity and the Pc tendency to aggregate. A number of possible delivery strategies have been suggested, ranging from the use of oil-in-water (o/w) emulsions to liposomes and nanoparticles as potential carrier vehicles reviewed in Josefson, et al [23, 39, 40, 41, 42, 43, 44] and to enhance the cellular uptake and subsequent antitumor efficacy of PDT [45, 46, 47].

Addition of Metformin to the GaPc-PDT treatment increased the therapy efficiency in both cell lines, similarly to our previous report for Walker carcinosarcoma PDT [7]. In the M1-15 metastatic melanoma, Metformin significantly increased PDT cell killing and induced apoptosis of the melanoma cells. However, the beneficial effect was relatively small in terms of viability decrease, when PDT was concerned; GaPc-PDT and Metformin increased the induction of apoptosis at a small dose of photosensitizer and light exposure.

The origin of the melanoma metastatic cells seems to be from a population of melanoma initiating cells, that resemble stem cells characters and are resistant to chemotherapy [48, 49]. These tumors stem cells are CD133 positive and induce chemoresistance by activating different survival proteins involved in the Akt/PKB and Bcl-2 pathway. Furthermore, activation of Akt/PKB pathway induces cellular antiapoptotic effects, increases protein synthesis and proliferation through rapamycin (mTOR) binding [50]. Metformin inhibits malignant metastatic and stem cell growth through blocking of the metabolic pathway AKT/mTOR. These effects were reported on different cell lines: hMCF-7 human mammary carcinoma and FSaII mouse fibrosarcoma cells [51] and combined with doxorubicine on *in vivo* and *in vitro* breast cancer tumors [52].

In cancerous cells, glucose metabolism is switched to aerobic glycolysis -Warburg effect, and generates a high amount of energy, as well as metabolites like lactate and ketones that promote tumor cells “immortality”. Metformin interferes with the mitochondrial metabolism and can lower both energy and glucose metabolites production [51]. These mechanisms might explain the addictive effect of Metformin to PDT efficiency.

In both cell lines, cell death correlated with the alteration of the cytoskeleton filaments and a high level of lipid peroxidation, indirect hallmarks of ROS, as a result of the Metformin induced inhibition of the antioxidant defense in melanoma cells. This is consistent with previous reports about the roles of PDT induced ROS leading to DNA damage and subsequent caspase activation followed by tumor cell death [53]. Cytoskeleton disorganization as a result of PDT has been reported in different cell lines: in a chronic myelogenous leukemia-derived cell line (K562), glioblastoma (D54Mg) in ALA mediated PDT, or WM35 in PDT with two mesoporphyrins meso-5,10,15,20-tetrakis (4-hydroxyphenyl) porphyrin (THOPP) and meso-5-(4-hydroxyphenyl)-10, 15, 20- tris (4-methoxyphenyl) porphyrin (THOMPP) [24, 54, 55]. PDT with zinc(II)-phthalocyanine (ZnPc) of three cell lines indirectly induced changes in microtubules and F-actin in HeLa cells, correlated with apoptosis [56].

In the current study we measured the protein levels of two members of the TNF family: TNF-α and TRAIL, secreted by the melanoma cells as a result of GaPc-PDT w/o Metformin, in an effort to understand their role in the switch between survival, leading to resistance to therapy and apoptosis in the melanoma cells. Similarly, with ROS, TNF-α has a dual role in melanomagenesis and response to therapy. Through binding to specific receptors, TNF-α can induce more than 5 pathways that end up with inflammation, apoptosis, proliferation, invasion, angiogenesis, metastasis or morphogenesis. Furthermore, these pathways lead to contradictory effects: both anti-apoptotic and pro-apoptotic [57]. Although, TNF-α is able to induce tumor cell death, its primary role is pro-inflammatory [58]. TNF-α is a negative prognostic factor in surgery and correlates with resistance to chemotherapy, while high tumor levels of TNF-α might be beneficial for the melanoma immunotherapy [44]. Melanoma WM35 cell line was previously found to be resistant to TNF-α but not to TRAIL induced apoptosis [59]. On the other hand, TRAIL selectively induces apoptosis of tumor and premalignant cells, but not normal cells [54]. Unfortunately, most primary cancer cells are resistant to TRAIL induced apoptosis due to preexistent p53 mutations, absence of specific death receptors from cell surface or presence of abundant decoy receptors [54]. TRAIL is involved in triggering the extrinsic pathway of apoptosis. Binding of TRAIL to surface death receptors initiates their trimerization and starts recruiting Fas-associated death receptor (FADD) leading to caspase-8 activation [54]. In type I cells, caspase-8 activation is enough for apoptosis, while, in type II cells, the extrinsic pathway should be completed with mitochondrial pathway activation to sustain the apoptotic process [60]. Expression of TRAIL receptor 2 on melanoma cells was correlated to a better prognosis [61].

In the WM35 melanoma cells, TNF-α as well as TRAIL expression and oxidative damage were strongly increased by the combined GaPc-PDT associated with Metformin, as opposed to single GaPc-PDT. In a previous PDT study, on WM35 cells, high levels of TNF-α generated by PDT were mainly anti-apoptotic and led to NF-kB activation, a mechanism involved in cell survival under oxidative stress [30]. In the current study, NF-kB expression, as well as the phosphorilated form was diminished, therefore, this mechanism of survival was not efficient in preventing oxidative cell death. Thus, the high expressions of both TNF family members led to the activation of apoptosis. The pro-apoptotic effects were strongly enhanced by Metformin treatment and are linked to the effects on mitochondrial glucose metabolism generated in the melanoma cells [51] that rendered the cells susceptible to a higher oxidative damage induced by PDT. This led to a combination of necrotic and apoptotic cell death. These effects were different in the highly metastatic M1-15 melanoma. M1-15 cells constitutively expressed high levels of TNF-α and TRAIL. This is probably due to the tumor promotion exerted by these molecules, by binding to pro-inflammatory receptors [62] and TRAIL decoy death receptors with roles in proliferation or decreased TRAIL sensitivity [57], [60, 62]. Combined exposure to Metformin and GaPc-PDT significantly decreased TNF-α, while endogenous TRAIL expression remained at high levels.

NO synthesis is mainly governed by iNOS, whose expression is regulated by the transcription factor NF-κB [34, 63, 64]. NF-κB protein was enhanced following GaPc-PDT, but the activation pathway was strongly inhibited by PDT, as proved by the decreased levels of IKκ and the phosphorilated form. The effect was increased by Metformin addition. As a result it also inhibited the expression of iNOS and, therefore, NO production, especially in M1-15 cells. On the other hand, NO may inhibit IKκ via S-nitrosylation, and decreases free NF- κB level, which creats a negative-feedback loop [65].

NF-κB and pNF-κB were slightly increased, following GaPc-PDT but this survival mechanism was not enough to prevent cell death [21, 44]. In these conditions, TRAIL induced apoptosis might be explained by the sensitization of the M1-15 melanoma to TRAIL, following the combined therapy. Sensitivity of tumor cells to TRAIL apoptosis may be restored by a number of substances and biological agents [61–66]: proteasome inhibitors, mainly, but not restricted to inhibition of NF-kB activation, AKT inhibition, mitogen-activated protein kinase (MAPK), protein kinase C (PKC) activation, reactive oxygen species, interferon, resveratrol, tunicamycin, histone deacetylase inhibitors, 2-methoxyestradiol, synthetic triterpenoids, peroxysome proliferators-activated receptor agonists, betulinic acid and telomerase-dependent virotherapy [61, 66]. Metformin exposure was involved in AKT inhibition [52, 53] and the combination with PDT increased therapeutically ROS induced damage, and decreased NF-κB activation. All these, were able to restore TRAIL sensitivity in M1-15 cells, leading to apoptosis. This behavior is consistent with a previous report, stating that inhibition of NF-κB in colon and mammary carcinoma cells switched the inflammatory LPS-induced tumor growth to tumor regression. This response was found to be independent of TNF-α, but dependent on TRAIL. Thus, an interesting future direction for increasing PDT efficiency in melanoma could be linked to exogenous TRAIL addition to this line of therapy, which has the potential to take advantage of the recovered TRAIL sensitivity in melanoma cells and to induce selective tumor cell apoptosis.

Both cell lines showed a low melanogenic level. Melanogenesis was inhibited by PDT in the metastatic M1-15 cells, while in the radial growth phase WM35, the effect was the opposite. Metformin seemed to have an inhibitory effect, while GaPc exposure increased melanogenesis. As such, combined treatment decreased melanogenesis activation due to the GaPc exposure. This is consistent with our previous PDT study, where we found increased melanogenesis following PDT with meso-substituted porphyrins on WM35 cells [29]. This effect was consistently seen on the total melanin content and the activity of the key melanogenic enzyme, tyrosinase. In WM35 cells, PDT induced a higher pigmentation level along with increased tyrosinase activity but it decreased the tyrosinase protein level. These differences can be explained by the activation of existing tyrosinase by PDT induced ROS [67]. However, ROS, especially hydrogen peroxide, in a high amount, may also be responsible for the inactivation of the tyrosinase [68]. Similarly, pigmentation could have been increased following PDT through ROS oxidation of melanin precursors. These differences were mainly seen in the WM35 cells, because of the low level of constitutive pigmentation that allowed the smaller modifications to became apparent. In the case of WM35, PDT also induced necrotic cell death, at a lower lipid peroxidation level compared to M1-15, possibly due to a lower melanoma resistance to oxidative stress. In patients with melanoma, the prognosis was shown to be influenced by the pigmentation. In patients with early stage (I and II) melanoma, pigmentation was correlated with a better survival, while in advanced stages, melanoma pigmentation impaired the prognosis [69]. This is probably due to the melanogenesis process that can delay tumor growth when is normal, while deregulation of pigment synthesis and/or pheomelanogenesis will have a stimulatory impact on tumor growth because of active melanin precursors, leading to ROS and accumulating mutations [69]. TRPM1 (melastatin) expression loss occurred at the transition of radial to vertical growth primary cutaneous melanoma [70]. Moreover, melanogenesis in metastatic melanomas was related to resistance to radio [71] and chemotherapy and also immunosuppressive effects [72], while the inhibition of melanogenesis restored melanoma sensitivity to gamma rays [73], cyclophosphamide and reverted the immunosuppression due to melanin synthesis [72]. Therefore, inhibition of melanogenesis is likely to improve the outcome of the therapy of metastatic melanoma [71]. Therefore, inhibition of melanogenesis, mostly seen in the metastatic M1-15 cell line represents a beneficial effect of the GaPc PDT, enhanced by Metformin addition.

Many factors were involved in stimulation of melanogenesis after UV exposure, like lipids peroxidation, ROS production, MAPK, TNF-α pathway, DNA damage—DNA repair processes enhanced by melanin [74, 75, 76]. Moreover, MITF expression was related to increased survival following oxidative stress [77]. Increased MITF was related to a lower prognosis in melanoma patients, resistance to therapies and particularly PDT, while decreased levels were associated with a better PDT therapeutic response [31]. Since MITF is very widely expressed in human melanomas, the found that the combination of PDT and Metformin had the ability to inhibit it, along with the inhibition of NF-κB pathway activation seems really promising. Moreover, this was consistent in both melanoma cell lines.

Neoangiogenesis is involved in tumor promotion, invasion and metastasis, critical steps in melanoma progression. Thus, we investigated the effects of GaPc-PDT and Metformin on the synthesis and expression of the main two factors involved in stimulating carcinomatous neoangiogenesis: VEGF and HIF-1α. HIF-1α is the transcription factor in charge of the cellular response to hypoxia, including generated by a growing malignant tumor environment that consists mainly in angiogenesis and apoptosis. Once stimulated, HIF-1α translocates into the nucleus, binds to the hypoxia-response element, and up-regulates the expression of VEGF and other angiogenic factors at mARN and protein levels [78, 79].

Our data showed that PDT and respectively Metformin and PDT inhibited the expression of the VEGF and HIF-1α, in the melanoma cells. However, future in vitro studies on co-culture models and *in vivo* studies are necessary to support these findings [80]. There is a strong, GaPc-PDT induced inhibition of VEGF and HIF-1α, consistent for both cell lines, while combined regimen decreased this effect. Previously, we have reported that the combination of Metformin and PDT reduced angiogenesis *in vivo*, on Walker carcinosarcoma [7]. There are reports showing a beneficial role of Metformin in the inhibition of neoangiogenesis [81, 82, 83]. Metformin also induced increased survival, accelerated tumor growth and increased VEGF-A in BRAF V600 mutant melanomas [84]. This is consistent with our findings of stimulating effects of Metformin on VEGF and HIF-1α in WM35 melanoma, since this cell type exhibits the *BRAF*-V600E-mutation. Interestingly, in the *BRAF*-V600E-mutated melanomas, it was reported that association of Metformin with anti-angiogenic therapies has a cumulative effect on cell killing [85]. In this respect, the combination of GaPc-PDT, with an inhibitory effect on angiogenesis and Metformin might explain the strong beneficial effect of cell death induction in WM35 cells. It was shown that HIF-1α is a direct target gene of NF-κB under non-hypoxic and certain hypoxic conditions. Activation of NF-κB leads to increased HIF-1α protein levels. HIF-1α also contributes to the activation of the NF-κB pathway; moreover, both proteins seem to be regulated by the same pathways, involving IKKβ hydroxylation [86]. This is consistent with our data, where there is a similar trend within these protein levels. Metformin was described to inhibit NF-κB activation in normal cell lines [35,87] senescent cells [88] and cancer cells [89]. The main antitumor mechanism seems to be related to reversible inhibition of the mitochondrial complex I function which has also been involved in the hypoxic inhibition of HIF-1α. The effect requires an intact mitochondrial inner membrane potential. Metformin reduced HIF-1α in colon cancer cells-HCT 116 p53^−/−^ and significantly diminished downstream HIF dependent proteins like VEGF, and carbonic anhydrase 9 (CA9) in these cells, under hypoxic conditions (1.5% O_2_), however, it slightly increased HIF-1α, VEGF and CA9 under normal O_2_ concentration (21%)[87].

The aim of our study was to find if Metformin might increase melanoma cells sensitivity to PDT and improve the overall treatment response. Therefore, in our experimental setting, cells with prior 24h Metformin exposure were exposed to GaPc for 24h, irradiated then collected after an additional 24h period, following PDT irradiation. Cells exposed to Metformin were treated with medium in a similar manner. Since the Metformin inhibition of mithocondrial function is reversible [87], the time elapsed between exposure and the end of the experiments diminished the Metformin effects and allowed the cells to recover, in the absence of PDT. This could explain the reactive increase of NF-κB activation, HIF-1α and VEGF in groups without PDT. When combined treatment was used, the angiogenetic markers like VEGF and at a lower extent HIF-1α, although significantly inhibited compared to controls, remained at higher levels, compared to PDT alone. This could be partially explained by the experimental setting, as above, however, this effect might be also due to the stimulatory effect of Metformin on these molecules in normoxic conditions [87]. In melanoma, induction of melanogenesis was shown to increase HIF-1α and stimulate the expression of several downstream HIF-1 regulated genes, including VEGF. This was attributed to the role of melanogenesis intermediates that lead to ROS and consumption of intracellular oxygen due to the activation of the melanogenic pathway [90]. These are consistent with our data for the GaPc treated cells, where increased HIF-1α, was correlated with high melanogenesis. When GaPc PDT was used, cells exhibited higher HIF-1α in WM35 cells that correlated with the absence of melanogenesis inhibition (lower melanin, but tyrosinase activity was similar to controls). In M1-15 cells, lower HIF-1α correlated with inhibition of melanogenesis.

PDT is an ideal oncological approach because induces direct tumor cell photo damage and also targets tumor vasculature and activates the immune response [91, 92]. Despite these effects, there is a limited experience of PDT in melanoma and extensive clinical studies need to be taken into consideration, using selected photosensitizers and standard irradiation protocols and also several combination regimens with chemotherapy or immunotherapy, in order to obtain an established antitumor effect [11].

## Conclusions

GaPc proved an efficient PS in PDT against these low pigmented melanoma cell lines: WM35 and M1-15. The beneficial antimelanoma effects of Metformin addition to PDT resided mainly in increased tumor cell killing through enhanced oxidative damage and induction of proapoptotic mechanisms, while the inhibition of tumor angiogenesis promoted by PDT was decreased by Metformin.

## Abbreviations

AKT/PKB: phosphatidylinositol 3-kinase and protein kinase B
Bcl-2: B cell lymphoma 2
BRAF: protooncogene B-Raf
DMSO: dimethylsulfoxide
DNA: deoxiribonucleic acid
DRAQ5: far red fluorescent DNA dye
ELISA: enzyme-linked immunosorbent assay
FACS: flowcytometry
FADD: Fas-associated death receptor
FITC: fluorescein isothiocyanate
GaPc: Gallium phthalocyanine chloride
GAPDH: glyceraldehyde-3-phosphate dehydrogenase human
HIF-1α: hypoxia inducible factor 1 alpha
IC50: Inhibitory concentration 50%
ikK: inhibitor of nuclear factor kappa-B-kinase
iNOS: nitric oxide synthase inducible
LPS: lipopolysaccharide
MAPK: mitogen-activated protein kinase
mARN: messenger ribonucleic acid
MDA: malondialdehyde
MITF: microftalmia transcription factor
mTOR: mammalian target of rapamycin
NF-κB: nuclear transcription factor kB
NO: nitric oxide
Pc: phthalocyanines
PDT: photodynamic therapy
PI: propidium iodide
PKC: protyein kinase C
pNF-κB: phosphorilated nuclear transcription factor kB
PS: photosensitizer
ROS: reactive oxygen species
RPMI: Roswell Park Memorial Institute
SDS PAGE: dodecyl sulfate polyacrylamide gel electrophoresis
TNF-α: tumor necrosis factor alpha
TRAIL: TNF-related apoptosis-inducing ligand
VEGF: vascular endotelial growth factor
WB: western blotting
WM1/15: metastatic cell line
WM35: melanoma in radial growth phase
